# Considerations for studying transmission of antimicrobial resistant enteric bacteria between wild birds and the environment on intensive dairy and beef cattle operations

**DOI:** 10.7717/peerj.6460

**Published:** 2019-02-27

**Authors:** Kristin Tormoehlen, Yvette J. Johnson-Walker, Emily W. Lankau, Maung San Myint, John A. Herrmann

**Affiliations:** 1 Department of Veterinary Clinical Medicine, University of Illinois at Urbana-Champaign, Urbana, IL, USA; 2 Department of Animal Sciences, University of Illinois at Urbana-Champaign, Urbana, IL, USA; 3 Ronin Institute, Montclair, NJ, USA; 4 Department of Animal Sciences, University of Wisconsin-Madison, Madison, WI, USA

**Keywords:** Antimicrobial resistance, Enteric bacteria, Wild birds, Microbial ecology, Transmission, *Escherichia coli*, *Enterococcus*

## Abstract

**Background:**

Wild birds using livestock facilities for food and shelter may contribute to dissemination of enteric pathogens or antimicrobial resistant bacteria. However, drivers of microbial exchange among wildlife and livestock are not well characterized. Predisposition for acquiring and retaining environmental bacteria may vary among species because of physiologic or behavioral differences, complicating selection of a bacterial model that can accurately characterize microbial connections among hosts of interest. This study compares the prevalence and antibiotic resistance phenotypes of two potential model bacterial organisms isolated from wild birds and their environments.

**Methods:**

We compared prevalence and resistance profiles of *Escherichia coli* and *Enterococcus* species isolated from environmental swabs and bird feces on a residential control site, a confinement dairy, a pasture-based beef farm, and a confinement beef farm.

**Results:**

Bird feces at all sites had low-to-moderate prevalence of *Escherichia coli* (range: 17–47%), despite potential for exposure on farms (range: 63–97%). Few *Escherichia coli* were isolated from the control environment. *Enterococcus faecalis* was dominant in birds at both beef farms (62% and 81% of *Enterococcus* isolates) and low-to-moderately prevalent at the dairy and control sites (29% and 23% of isolates, respectively). Antimicrobial resistance prevalence was higher in farm samples compared to those from the residential control, but distribution of resistant isolates varied between the bacterial genera. Birds on all farms carried resistant *Enterococcus* at similar rates to that of the environment, but resistance was less common in bird-associated *Escherichia coli* despite presence of resistant isolates in the farm environment.

**Discussion:**

Bacterial species studied may affect how readily bacterial exchange among populations is detected. Selection of microbial models must carefully consider both the questions being posed and how findings might influence resulting management decisions.

## Introduction

Bacteria are transferred among farms through complex networks (Arnold, Williams & Bennett, 2016). Wildlife are a component of these networks that could contribute to transmission of pathogens or antimicrobial resistance between facilities due to potentially high mobility and seasonal movement patterns (Greig et al., 2015; Arnold, Williams & Bennett, 2016). While wildlife living in farm environments are recognized participants in microbial exchange on farms, the dynamics of bacterial exchange among species in the farm environment are poorly understood (Arnold, Williams & Bennett, 2016).

Wild birds may frequent farm environments in large mixed-species flocks due to readily available food, shelter, and roosting or nesting sites. While wild birds have been documented to carry a variety of potentially important bacterial pathogens and antimicrobial resistant bacteria (Brittingham, Temple & Duncan, 1988; Abulreesh, Goulder & Scott, 2007; Greig et al., 2015; Arnold, Williams & Bennett, 2016), in many cases, neither the directionality and frequency of transmission, nor clinical significance of these findings for herd management are clear (Greig et al., 2015; Arnold, Williams & Bennett, 2016). Farmers apply a variety of management approaches to control or reduce bird populations on the farm (US Department of Agriculture, 2015), that have unknown effects for mitigating potential bird contributions to microbial transmission.

Most studies addressing bacterial exchange among birds and livestock have focused on a few clinically important bacterial species, specifically pathogenic or antimicrobial drug resistant *Escherichia coli*, *Salmonella enterica*, *Campylobacter* species, and to a lesser extent, *Enterococcus* species (Arnold, Williams & Bennett, 2016; Vittecoq et al., 2016). These studies are valuable for understanding the microbial ecology and epidemiology of these pathogens but may or may not be generalizable for understanding microbial exchange dynamics in the farm environment more broadly.

Geographic and temporal patterns in genetic or phenotypic markers of shared commensal bacterial species can support causal reasoning about transmission direction or dynamics and can guide management decisions. Recent studies have applied analyses of molecular genetics and antimicrobial resistance patterns to building causal models of microbial exchange between wildlife and human or livestock populations (Goldberg et al., 2007; Rwego et al., 2008; Wheeler et al., 2012; Pesapane, Ponder & Alexander, 2013; VanderWaal et al., 2013). Selection of *Escherichia coli* as a model organism in most of these studies is primarily a choice of convenience due to ease of laboratory cultivation and well-characterized ecology and genetics, but this may not be the optimal model for studying microbial connectivity in all wildlife species of interest and might not reveal the same patterns as other members of the gut microbiota.

For example, Galapagos iguanas have unique feeding ecology and associated diverse microbiomes well-suited to support fermentative digestion (marine algae in the case of marine iguanas and terrestrial plant materials in the case of land iguanas; Hong et al., 2011). Both community sequencing and cultivation-based assessment suggest that *Escherichia coli* was a relatively uncommon or low-abundance resident of the gastrointestinal tracts of Galapagos iguanas relative to other gram-negative species. *Escherichia coli* was only cultured regularly from individuals captured near high-densities of humans (Hong et al., 2011; Wheeler et al., 2012). While antimicrobial resistance was detected in *Escherichia coli* isolated from human-proximate iguana populations, *S. enterica* from those same individuals rarely demonstrated antimicrobial resistance, demonstrating that the bacterial species studied can substantially impact the conclusions of studies seeking to understand the potential for antimicrobial resistance or pathogen spread by free-ranging wildlife species (Wheeler et al., 2012). While a gradient of human influence on *Escherichia coli* resistance traits was detected in the Galapagos system, *Escherichia coli* appeared to be a relatively uncommon, possibly transient member of Galapagos iguana enteric biota such that this study may have underestimated the potential for bacterial exchange between these species and human population centers due to bacterial model selection (Wheeler et al., 2012). A limited number of studies support the notion that wildlife microbiomes can be quite resistant to bacterial species incursion from changing environmental exposures, but this resistance may vary by host species or bacterial taxa (McCord et al., 2013; Risely et al., 2017a, 2017b) such that some bacterial species may be more easily or frequently exchanged with environmental sources than others.

Thus, limited understanding of wildlife microbiomes may curb efforts to assess the true magnitude of wild bird contributions to bacterial dissemination. While *Escherichia coli* is a common commensal of many species, it may not be the ideal model for studying transmission among those with wide taxonomic or ecological distinction from livestock, poultry, or humans. Understanding of the microbiomes of non-mammalian taxa has lagged (Colston & Jackson, 2016). Bird, reptile, amphibian, and fish microbiota share broad characteristics with mammals, but the relative abundance of dominant groups and specific species memberships vary by factors such as taxonomy, diet, and environmental exposures (Waite & Taylor, 2014; Colston & Jackson, 2016).

In farm settings, birds and other wildlife are potentially exposed to many bacterial species and strains but these exposures may or may not result in acquisition, retention, or shedding of specific bacterial species such that different models could provide quite different estimates of dissemination rates. The goal of this pilot study was to consider *Escherichia coli* and *Enterococcus* species, particularly *Enterococcus faecalis*, as possible model systems for studying transmission dynamics in wild birds in livestock farm environments. Specifically, we sought to understand whether these models demonstrated similar associations between birds and environmental bacterial pools across sampling sites using antimicrobial resistance profiles as a low-cost phenotypic marker.

## Materials and Methods

### Sampling sites and methodology

Sampling took place during the fall of 2008 and summer of 2009. Four sites were selected for this study: one non-farm location in a rural residential neighborhood (hereafter called the “Control”) and three livestock production facilities. The Control was in a rural residential area greater than five miles from any known large livestock or poultry production sites. The three livestock production sites were located on the campus of the University of Illinois at Urbana-Champaign. These sites were selected for proximity to laboratory resources, ease of access, and habitat diversity to assess how the two bacterial genera compared in farm environments with different opportunities for bird-livestock interaction. They consisted of a 400 head confinement dairy farm (Dairy), a pasture-based beef cow and calf farm (Beef A), and a confinement beef cow and calf farm (Beef B). Beef A and Beef B collectively housed 750 head, which were rotated between the two facilities. The Dairy and Beef A facilities were less than 0.25 miles apart, whereas Beef B was located just over a mile southeast of the other facilities. The Control was located approximately 12 miles southeast of the university in Sydney, Illinois. The Dairy and Beef B sites were sampled during the fall of 2008 and the Control and Beef A sites were sampled during the summer of 2009.

A convenience sampling technique was used to obtain individual animal samples from free-living birds at each site. Birds were captured using mist nets and two swabs (feces, or if not available, cloacal swabs) were collected from each bird. The nets were placed in areas where flocks of birds were observed to fly through the sites when possible but, were limited to marginal areas of the farm environments so as not to interfere with farm operations. Nets were set up in the early morning for 3–4 h and again in the late afternoon for 3–4 h. To prevent repeat sampling of birds, the tip of a tail feather was clipped prior to release as a temporary identification for previously sampled birds over the 2–4 days of trapping performed at each site. The target sample size of 30 per site was determined using standard sample size calculation formulae to achieve a 95% sensitivity for a survey assuming 100% test specificity using the binomial method. Underlying assumptions were 10% prevalence, 98% test sensitivity, 95% desired population sensitivity, and unknown total population size (Humphry, Cameron & Gunn, 2004). All capture and handling were performed as a sub-permitee of the Illinois Natural History Survey’s master banding permit (#06507) and under an approved animal care and use protocol with associated biosafety oversight through the University of Illinois (IACUC protocol #08114).

Environmental samples from each site were collected via drag swab. At the three cattle farms, drag swab samples were obtained from the ground or flooring in cow gathering areas where birds were observed (i.e., dry lot, barns, milking parlor entrance, pasture, feed, and watering areas) including any freshly voided manure that may have been present. Two swabs were taken from each area sampled. At the control site drag swabs were collected in the same manner from outdoor surfaces (e.g., bird feeder platform, sidewalk, lawn, bird bath) that were accessible to free-living wild birds.

Between 60 and 68 samples were collected from each livestock agricultural site, 30 bird and 30 cattle environment samples from Beef A and Beef B and 38 bird and 30 cattle environment samples from the Dairy site. At the control site, 50 samples were collected, 30 birds and 20 environmental samples (fewer control environmental samples were collected due to smaller land area of the site allowing for representative sampling with fewer swabs). All 238 samples were labeled and transported on cold packs to the laboratory for bacterial isolation and analysis within 12 h of collection.

### *Escherichia coli* cultivation

Gram-negative bacteria were isolated using standard cultivation techniques for each species (Murray et al., 2007). One swab from each bird or environmental source was first placed in 10 ml of buffered peptone water (Difco Laboratories, Detroit, MI, USA) and was incubated at 37 °C for 24 h. Following incubation, a loop of turbid buffered peptone water was streaked for isolation on MacConkey agar (Difco Laboratories, Detroit, MI, USA) and incubated at 37 °C for up to 48 h. A well-isolated colony on MacConkey agar was then re-streaked for isolation on eosin methylene blue agar (i.e., a single isolate was collected per sample) BD Diagnostic System, Franklin Lakes, NJ, USA) and incubated for 24–48 h at 37 °C. Presumptive *Escherichia coli* colonies on eosin methylene blue agar were confirmed as *Escherichia coli* if negative for citrate metabolism on Simmons citrate agar (Thermo Fisher Scientific, Waltham, MA, USA) and positive for indole production in tryptophan broth (Thermo Fisher Scientific, Waltham, MA, USA). Confirmed isolates were stored on nutrient agar (Thermo Fisher Scientific, Waltham, MA, USA) for additional testing.

### *Enterococcus* species cultivation and identification

*Enterococcus* species were isolated using standard microbiological techniques (Murray et al., 2007). Briefly, each swab was placed into two ml of *Enterococcus* selective bile-esculin broth (Thermo Fisher Scientific, Waltham, MA, USA) and incubated at 40 °C for 24 h as a pre-enrichment step. Bile-esculin broths that turned dark brown to black during incubation, suggesting the presence of *Enterococcus* and related species, were then streaked for isolation onto m-*Enterococcus* agar (Thermo Fisher Scientific, Waltham, MA, USA) and incubated at 35 °C for 24 h. Plates were incubated for up to 48 h before determining no growth was present. A single isolate was selected for identification and antimicrobial resistance testing.

Membership in the genus *Enterococcus* was confirmed for each isolate obtained using standard metabolic tests. Isolates that were catalase positive, positive for esculin hydrolysis on bile-esculin agar, and grew in brain-heart infusion broth with 6.5% NaCl were considered confirmed *Enterococcus* isolates. Isolates were then identified to species using a multiplex PCR protocol targeting the superoxide dismutase gene (Jackson, Fedorka-Cray & Barrett, 2004). Any isolates that could not be identified using this protocol were submitted for sequencing of the manganese-dependent superoxide dismutase gene (Poyart, Quesnes & Trieu-Cout, 2000).

### Antimicrobial susceptibility testing

Established protocols were used for determination of antimicrobial susceptibility (Galland et al., 2001; National Committee for Clinical Laboratory Standards (NCCLS), 2003). All samples were streaked onto nutrient agar from refrigerated stocks and incubated at 37 °C for 24 h prior to testing. Two to three isolated colonies were added to two ml of Luria-Bertani broth and incubated at 37 °C on a Innova 2300 platform shaker (New Brunswick Scientific, Edison, NJ, USA) at 200 rpms for 30 min until turbidity in each tube matched the turbidity standard (0.5 McFarland). Then a swab was saturated with the broth and streaked onto a Mueller-Hinton agar plate (Becton, Dickinson and Company, Franklin Lakes, NJ, USA). Antimicrobial drug discs (BD BBL™ Sensi-Disc™ Susceptibility Test Discs, Becton, Dickinson, and Company, Franklin Lakes, NJ, USA) were placed on the plate and the plate was incubated at 37 °C for 24 h prior to measuring the zones of inhibition (diameter in millimeters) with a digital caliper. *Escherichia coli* were tested for susceptibility to amoxicillin with clavulanic acid (20/10 μg), ampicillin (10 μg), azithromycin (15 μg), ceftiofur (30 μg), cephalothin (30 μg), chloramphenicol (30 μg), rifampin (five μg), sulfamethoxazole-trimethoprim (23.75/1.25 μg), and tetracycline (30 μg). All confirmed *Enterococcus* genus isolates (regardless of species) were tested for susceptibility to erythromycin (15 μg), gentamicin (10 μg), penicillin G (10 units), sulfamethoxazole-trimethoprim (23.75/1.25 μg), tetracycline (30 μg), and vancomycin (30 μg) (see also Table S1). Antimicrobials were selected for each bacterial species based on antimicrobial spectrum, common use for clinical treatment of cattle, or pertinence to public health concerns about agriculture-associated antimicrobial drug resistance (in particular, vancomycin). Laboratory strains of *Escherichia coli* (ATCC 25922) and *Enterococcus faecalis* (ATCC 29212) were analyzed with each set of field isolates as quality control standards for the disc diffusion assay.

### *Escherichia coli* genotyping

To explore the degree of isolate pool overlap among locations given the proximity of the sampling sites, *Escherichia coli* were genotyped by repetitive element palindromic PCR, which detects the distribution of repetitive DNA sequences as genomic fingerprints (Versalovic, Koeuth & Lupski, 1991; Rademaker et al., 1998). *Escherichia coli* isolates were re-streaked for isolation from the refrigerated stocks onto nutrient agar and incubated at 37 °C for 24 h. A single isolated colony was added to 100 μl of sterile deionized water to make a template for PCR amplification using primers ERIC1R (5′-ATG TAA GCT CCT GGG GAT TCA-3′) and ERIC2 (5′-AAG TAA GTG ACT GGG GTG AGC G-3′) targeting enterobacterial repetitive intergenic consensus repetitive motifs. Reactions were performed on a TGradient thermocycler (Biometra, Göttingen, Germany). The PCR program was as follows: denaturation at 95 °C for 2 min followed by 30 cycles each of 94 °C for 3 s, 92 °C for 30 s, and 50 °C for 1 min and a final extension at 65 °C for 8 min. Polymerase chain reaction amplification mixtures (25 μl) included 1.75 U of Takara Taq polymerase (Takara Bio Inc., Kusatsu, Japan), 1X Takara PCR Buffer with 2.5 mM (final concentration) of MgCl2, 2.0 mM Takara dNTP mixture (0.5 mM each), 1 μM each of the forward and reverse primers, and approximately 50 ng of template. PCR products were separated on a 2% Agarose gel in 0.75% Tris-Acetate EDTA run at 80 V for 12 h. Three lanes of a one kb DNA ladder (Genlantis, San Diego, CA, USA) were included to allow for standardization of molecular weight assignments to DNA fragments. Gels were stained with ethidium bromide and digitally photographed using an AlphaImager ™ 2200 (Alpha Innotec Co., San Leandro, CA, USA). *Enterococcus* isolates were not genotyped at the time of collection due to resource limitations and are no longer available for testing at the time of this writing.

### Statistical analysis

Antimicrobial drug susceptibility profiles were recorded as zone of inhibition measurements in millimeters and were then assigned to susceptible, intermediate, or resistant phenotypes for categorical analysis of susceptibility patterns using established clinical breakpoints provided in the antimicrobial drug disc product insert or published literature (Oxoid Antimicrobial Susceptibility Discs, Thermo Scientific, Waltham, MA, USA; Burton et al., 1996; Table S1). Clinical resistance thresholds are not defined for rifampin in members of Enterobacteriaceae. The normal diameter range for quality control using *Escherichia coli* ATCC 25922 (8–10 mm) was used to indicate isolates with near absolute resistance to rifampin vs organisms with larger zones of inhibition, however, these arbitrary thresholds do not have clinical relevance for treatment as rifampin is generally not recommended for *Escherichia coli* infections. The intention of using clinical breakpoints for classifying antimicrobial drugs in this analysis was for phenotypic categorization of isolates, not interpretation of whether or not treatment with these medications would be effective in a clinical infection with the isolates. Breakpoints for *Enterococcus* species are ≤16, 17–19, and ≥20 mm for resistant, intermediate, and susceptible, respectively, whereas all *Escherichia coli* isolates examined in this study had zones of inhibition less than 17 mm (range: growth adjacent to the disc at ∼7–17 mm).

Data analysis was performed in R version 3.2.1 (R Core Team, 2017). Comparisons between categorical variables was performed using either a two-sided Chi-square or Fisher’s exact test (when assumptions for the former were not met) with simulated *p*-values using the Chi-square test or Fisher’s exact test functions in the stats package of R, respectively.

Generalized linear models were used to identify factors associated with the presence of antimicrobial resistance (binomial model with presence or absence of resistance to one or more of the antimicrobial drugs tested as the outcome) and severity of resistance (negative binomial model with count of antimicrobial drugs to which each isolate demonstrated resistance as outcome variable). Sampling site (Control, Dairy, Beef A, and Beef B), sample source (bird feces/cloacal swab or environmental swab), bacterial genus (*Escherichia* or *Enterococcus*), and *Enterococcus* species (*Enterococcus casseliflavus*, *Enterococcus durans*, *Enterococcus faecalis*, *Enterococcus faecium*, *Enterococcus gallinarum*, and *Enterococcus hirae*), along with interaction terms, were considered as explanatory variables for antimicrobial resistance patterns. Logistic models (resistance presence-absence) were run using the glm function in the stats package in R statistical language (R Core Team, 2017) and negative binomial models (resistance severity) were run using the glm.nb function in the MASS package in R (Venables & Ripley, 2002). Both the resistance presence and resistance severity models were first run with all isolates included regardless of bacterial species (All isolate model). Separate models for *Escherichia coli* and *Enterococcus* species were then run to explore the significant interactions of sampling site and sample source with bacterial genus.

Rep-PCR band assignment and sizing from gel images was done using BioNumerics version 4.0 (Applied Maths, Sint-Martens-Latem, Belgium). Band assignments were exported as a binomial presence/absence matrix for statistical analysis. The Adonis function in the vegan package in R using the default Bray–Curtis distance (Oksanen et al., 2018) was used to perform a permutation or non-parametric multivariate analysis of variance analyses (MANOVA) to evaluate the effects of explanatory variables source (bird or environment), site (control, dairy, beef A, and beef B), and their interaction term on bacterial population structure. These models partition the sums of squares of distance matrices among treatments and have relaxed assumptions relative to traditional MANOVA. Significance in the permutation tests was determined by comparing the observed effects against 20,000 random permutations of the data for each model run independently.

Multivariate ordinations were then performed on the genomic banding patterns to visualize the significant effects detected by permutation MANOVA, using a constrained principal coordinates analysis, performed with the capscale function in the vegan package using a Jaccard distance (Oksanen et al., 2018). The ordination was performed on the full sample set using the interaction model of site and source as the constraining model and the bird and environmental sourced isolate subsets using sampling site as the constraining variable, as this factor explained the largest amount of variation (i.e., had the highest *R*^2^) in the various permutation MANOVA analyses.

## Results

### Sample description

We sampled 128 individuals of 13 bird species (Table S6) overall, house sparrows (48.0%), red-winged blackbirds (16.4%), European starling (14.8%), and brown-headed cowbirds (10.2%) made up the majority. Dominant species captured at the residential control site were house sparrows (40.0%) and mourning doves (33.3%). Birds captured on the Dairy facility were predominantly house sparrows (78.9%), on Beef A were predominantly European starlings (56.7%) and American robins (10.0%), and on Beef B were predominantly red-winged blackbirds (50.0%), house sparrows (20.0%), and brown-headed cowbirds (16.7%).

### Prevalence of enteric bacteria in wild bird feces and environmental swabs

Prevalence of the bacterial species differed between wild bird and environmental samples and among sites. *Escherichia coli* was commonly isolated from environmental samples in the three farm environments (site prevalence ranging 70.0–90.0% of samples, Table 1), and was significantly less frequently isolated from the residential control environment (pair-wise Chi-squared tests with control as the referent: *p* < 0.001 in all cases, Table 1). However, birds from all four sites carried *Escherichia coli* at low-to-moderate levels. Approximately a third of wild birds sampled were positive for *Escherichia coli*, with Beef B having the lowest *Escherichia coli* prevalence in wild birds (16.7% of 30 samples) and the Dairy site having the highest (47.4% of 38 samples; Table 1).

**Table 1:** Prevalence of two enteric bacterial genera in bird feces and environmental samples by site and isolate source.

Sample type	Site	*N*	*Escherichia coli* positive *N* (%)	Pair-wise *p*-value^&^	*Enterococus* spp positive *N* (%)	Pair-wise *p*-value^&^
Bird feces		128	47 (36.7%)		101 (78.9%)	
	Control	30	13 (43.3%)	Ref.	21 (70.0%)	Ref.
	Dairy	38	18 (47.4%)	0.807	24 (63.2%)	0.613
	Beef A	30	11 (36.7%)	0.792	27 (90.0%)	0.103
	Beef B	30	5 (16.7%)	0.046*	29 (96.7%)	0.012*
Environmental		110	77 (70.0%)		93 (84.5%)	
	Control	20	3 (15.0%)	Ref.	14 (70.0%)	Ref.
	Dairy	30	21 (70.0%)	<0.001*	26 (86.7%)	0.277
	Beef A	30	27 (90.0%)	<0.001*	27 (90.0%)	0.128
	Beef B	30	26 (86.7%)	<0.001*	26 (86.7%)	0.277

**Notes:**

& Pearson’s Chi-squared test or Fisher’s exact test when assumptions of the Chi-squared were not met; statistical tests were all run permutation tests with simulated *p* values and 10,000 iterations.

* Significant at α = 0.05.

*Enterococcus* species were also commonly isolated from the environment (site prevalence ranging 63.2–96.7% of samples, Table 1). Only Beef B differed in prevalence of *Enterococcus* species compared the control site (*p* = 0.012, Table 1). *Enterococcus* species were also commonly isolated from bird feces (site prevalence ranging from 70.0% to 96.7% of samples; Table 1). Eight species of *Enterococcus* were identified among the 194 isolates obtained from either bird feces or environmental swabs: *Enterococcus faecalis* (35.1% of *Enterococcus* isolates), *Enterococcus hirae* (28.9%), *Enterococcus casseliflavus* (14.4%), *Enterococcus faecium* (11.3%), *Enterococcus durans* (7.2%), *Enterococcus gallinarum* (2.1%), *Enterococcus haemoperoxidus* (<1%), and *Enterococcus avium* (<1%).

*Enterococcus hirae* (33.3% of 93 isolates) was the most common species isolated from environmental samples, followed by *Enterococcus faecalis* and *Enterococcus casseliflavus* (each 20.4% of 93 isolates). Different species were dominant at each site in the environmental samples: *Enterococcus hirae* and *Enterococcus faecalis* (each 42.9% of 14 isolates) at the residential control site; *Enterococcus faecium* (38.5% of 26 isolates) and *Enterococcus faecalis* (26.9% of 26 isolates) at the Dairy site; *Enterococcus hirae* (46.2% of 26 isolates) and *Enterococcus casseliflavus* (19.2% of 26 isolates) at Beef B; and *Enterococcus casseliflavus* (48.1% of 27 isolates) and *Enterococcus hirae* (33.3% of 27 isolates) at Beef A. In contrast, *Enterococcus faecalis* (48.5% of 101 isolates) was the most common species isolated from bird samples, followed by *Enterococcus hirae* (25.7% of 101 isolates). *Enterococcus hirae* was the dominant isolate from bird samples at the control site (67% of 21 isolates). *Enterococcus faecalis* was the most common *Enterococcus* species isolated from birds at both beef facilities and one of two equally common species at the Dairy site (Beef B: 62.1% of 29 isolates, Beef A: 81.5% of 27 isolates, and Dairy: 29.2% of 24 isolates along with *Enterococcus faecium*).

### Site and source patterns in the presence of antimicrobial drug resistance

Patterns in the presence of antimicrobial drug resistance differed among the four sites, by sample source, and by antimicrobial drug. Overall, 42.8% of all isolates demonstrated resistance to one or more antimicrobial drugs, with comparable percentages for *Escherichia coli* and *Enterococcus* isolates (40.5% and 44.4% of isolates, respectively). The percentage of isolates resistant to one or more antimicrobial drugs was higher in environmental isolates compared to those derived from birds (51.0% vs 33.3% for all isolates), but this difference was more pronounced for *Escherichia coli* isolates (52.9% vs 20.9%) compared to *Enterococcus* isolates (59.4% vs 39.8%).

**Figure 1:**
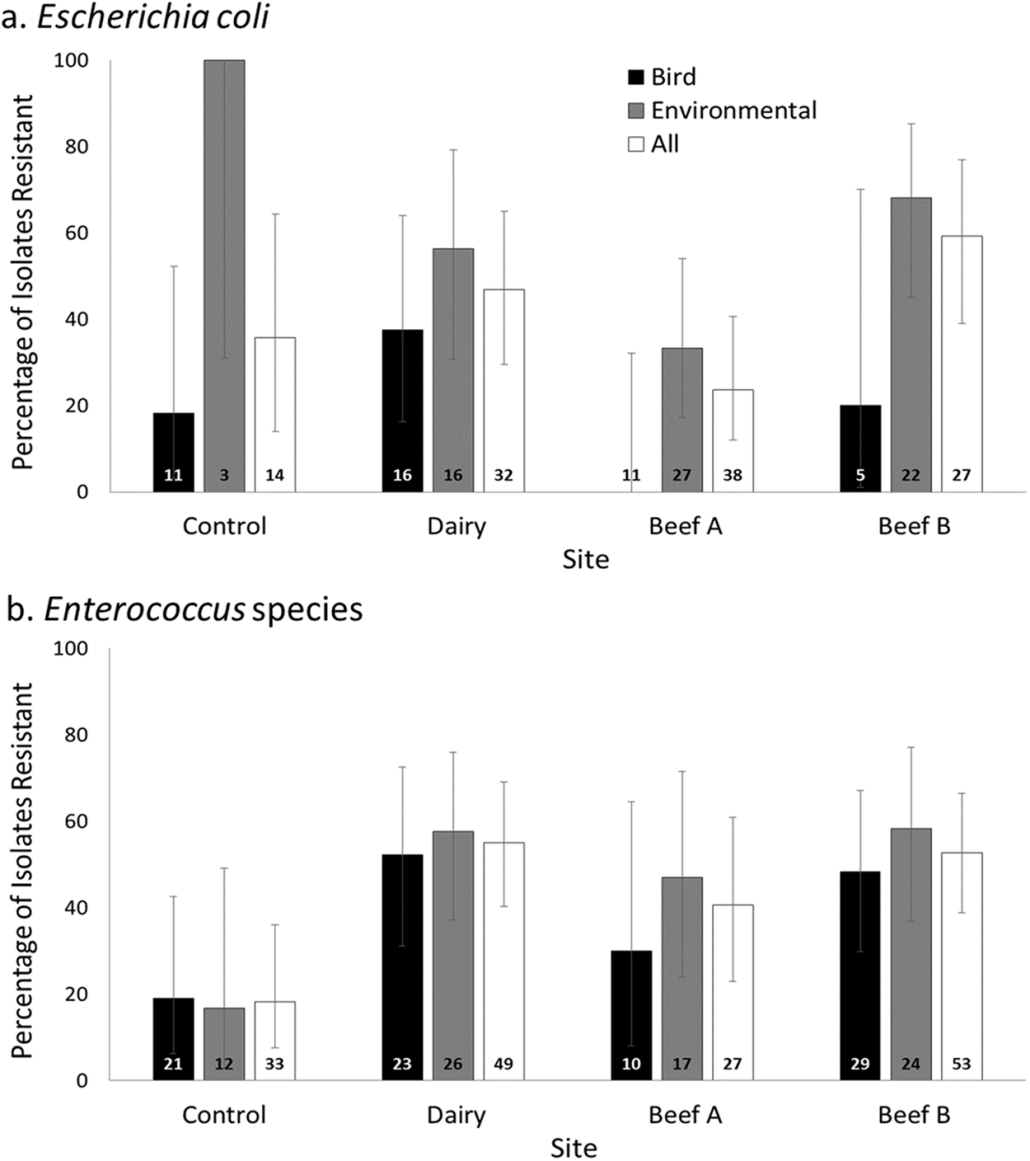
Prevalence of antimicrobial resistance by site, bacterial genus, and isolate source. Percentage (±95% CI of proportions; total *n* for each group provided at bar bases) of *Escherichia coli* (A) and *Enterococcus* (B) isolates resistant to one or more antimicrobial drug at a residential control and three cattle farm sites in central Illinois by sample source. The total proportions of *Escherichia coli* isolates resistant to one or more antimicrobial drug at each farm site did not differ significantly from the Control (Pairwise comparisons with Control as referent: all *p* > 0.05). *Enterococcus* isolates from the farm sites has significantly higher proportions of resistant isolates for two of three sites (Fisher’s exact, pair-wise comparisons with Control as referent: Dairy *p* = 0.001, Beef A *p* = 0.080, Beef B *p* = 0.0002).

For *Escherichia coli*, the majority of antimicrobial drug resistant isolates derived from environmental samples, with resistance less commonly detected in isolates from bird feces, with the exception of the Dairy (Fig. 1A). In contrast, the percentage of *Enterococcus* isolates resistant to one or more antimicrobial drugs tested was comparable for bird and environmental samples and the two of the three farms had significantly higher percentages of isolates demonstrating resistance (Fig. 1B).

Resistance in *Escherichia coli* was observed at variable levels to all antimicrobial drugs tested except for azithromycin (Table 2). *Escherichia coli* isolates demonstrating resistance to one or more the antimicrobial drugs tested were isolated from both birds and environmental samples at the Dairy, ranging from 3.1% (ceftiofur and amoxicillin) to 18.8% (tetracycline) of isolates overall, with generally comparable prevalence for each antimicrobial drug in birds and environmental samples. Resistant *Escherichia coli* were less commonly observed at both beef facilities, primarily in environmental samples (Table 2).

**Table 2:** Prevalence of antimicrobial drug resistance in *Escherichia coli* isolates by site and isolates source.

Site	Source	Trimethoprim-sulfamethoxazole*	Tetracycline	Cephalothin	Ceftiofur	Ampicillin	Rifampin	Amoxicillin	Azithromycin	Chloramphenicol
Control										
*n* = 11	Bird	1 (9.1%)	0 (0%)	0 (0%)	0 (0%)	0 (0%)	1 (9.1%)	0 (0%)	0 (0%)	0 (0%)
*n* = 3	Env	0 (0%)	0 (0%)	0 (0%)	0 (0%)	0 (0%)	3 (100%)	0 (0%)	0 (0%)	0 (0%)
*n* = 14	Total	1 (7.1%)	0 (0%)	0 (0%)	0 (0%)	0 (0%)	4 (28.6%)	0 (0%)	0 (0%)	0 (0%)
Dairy										
*n* = 16	Bird	0 (0%)	2 (12.5%)	1 (6.3%)	0 (0%)	2 (12.5%)	3 (18.8%)	0 (0%)	0 (0%)	1 (6.3%)
*n* = 16	Env	2 (12.5%)	4 (25%)	2 (12.5%)	1 (6.3%)	3 (18.8%)	7 (43.8%)	1 (6.3%)	0 (0%)	3 (18.8%)
*n* = 32	Total	2 (6.3%)	6 (18.8%)	3 (9.4%)	1 (3.1%)	5 (15.6%)	10 (31.3%)	1 (3.1%)	0 (0%)	4 (12.5%)
Beef A										
*n* = 11	Bird	0 (0%)	0 (0%)	0 (0%)	0 (0%)	0 (0%)	0 (0%)	0 (0%)	0 (0%)	0 (0%)
*n* = 27	Env	0 (0%)	3 (11.1%)	0 (0%)	0 (0%)	0 (0%)	6 (22.2%)	0 (0%)	0 (0%)	0 (0%)
*n* = 38	Total	0 (0%)	3 (7.9%)	0 (0%)	0 (0%)	0 (0%)	6 (15.8%)	0 (0%)	0 (0%)	0 (0%)
Beef B										
*n* = 5	Bird	0 (0%)	0 (0%)	0 (0%)	0 (0%)	0 (0%)	1 (20%)	0 (0%)	0 (0%)	0 (0%)
*n* = 22	Env	1 (4.5%)	6 (27.3%)	0 (0%)	0 (0%)	0 (0%)	10 (45.5%)	0 (0%)	0 (0%)	0 (0%)
*n* = 27	Total	1 (3.7%)	6 (22.2%)	0 (0%)	0 (0%)	0 (0%)	11 (40.7%)	0 (0%)	0 (0%)	0 (0%)
All (*N* = 111)		4 (3.6%)	15 (13.5%)	3 (2.7%)	1 (0.9%)	5 (4.5%)	31 (27.9%)	1 (0.9%)	0 (0.0%)	4 (3.6%)

**Note:**

* All columns show number resistant (% of n for each row).

A larger percentage of bird-derived *Enterococcus* isolates were resistant to antimicrobial drugs. As for *Escherichia coli,* resistance prevalence in birds generally reflected that found in the environmental isolates at the Control and Dairy sites, but in contrast to *Escherichia coli* resistance also reflected environmental samples at the beef and dairy sites (Table 3). Resistance was uncommon in *Enterococcus* isolates from the control site. Resistance to gentamicin (4.8% of 21 bird isolates and 8.3% of 12 environmental isolates) and to vancomycin (8.3% of 12 environmental isolates only) was observed at low prevalence at the control site (Table 3).

**Table 3:** Prevalence of antimicrobial drug resistance in *Enterococcus* isolates by site and isolate source.

Site	Source	Gentamicin *N* = 160*^,^**	Erythromycin *N* = 159*	Penicillin G *N* = 161*	Vancomycin *N* = 162	Trimethoprim-sulfamethoxazole *N* = 160*	Tetracycline *N* = 161*
Control							
*n* = 21	Bird	1 (4.8%)	0 (0%)	0 (0%)	0 (0%)	0 (0%)	3 (14.3%)
*n* = 12	Environmental	1 (8.3%)	0 (0%)	0 (0%)	1 (8.3%)	0 (0%)	0 (0%)
*n* = 33	Total	2 (6.1%)	0 (0%)	0 (0%)	1 (3%)	0 (0%)	3 (9.1%)
Dairy							
*n* = 23	Bird	3 (13.6%)	1 (4.8%)	2 (8.7%)	0 (0%)	1 (4.3%)	9 (39.1%)
*n* = 26	Environmental	2 (7.7%)	5 (20%)	1 (3.8%)	3 (11.5%)	1 (3.8%)	11 (42.3%)
*n* = 49	Total	5 (10.4%)	6 (13%)	3 (6.1%)	3 (6.1%)	2 (4.1%)	18 (36.7%)
Beef A							
*n* = 10	Bird	2 (20%)	3 (30%)	0 (0%)	0 (0%)	0 (0%)	3 (30%)
*n* = 17	Environmental	1 (5.9%)	4 (23.5%)	0 (0%)	0 (0%)	0 (0%)	7 (41.2%)
*n* = 27	Total	3 (11.1%)	7 (25.9%)	0 (0%)	0 (0%)	0 (0%)	10 (37.0%)
Beef B							
*n* = 29	Bird	7 (24.1%)	5 (17.2%)	0 (0%)	3 (10.3%)	1 (3.4%)	4 (13.8%)
*n* = 24	Environmental	0 (0%)	6 (25%)	0 (0%)	0 (0%)	0 (0%)	11 (47.8%)
*n* = 53	Total	7 (13.5%)	11 (20.8%)	0 (0%)	3 (5.7%)	1 (1.9%)	15 (28.8%)
All (*N* = 162)		17 (10.6%)	24 (15.1%)	3 (18.6%)	7 (4.3%)	3 (1.9%)	46 (28.6%)

**Notes:**

* Total number of isolates tested were less than the total number of isolates due to laboratory error during testing.

** All columns show number resistant (% of *n* for each row).

### Resistance to more than one antimicrobial drug

Resistance to more than one antimicrobial drug was observed in a small number of isolates from both genera. Overall, 10.2% of all isolates demonstrated resistance to more than one antimicrobial drug. A slightly higher percentage of *Enterococcus* isolates (12.3%) compared to *Escherichia coli* isolates (7.2%) were resistant to two or more medications. *Escherichia coli* isolates from bird feces had the lowest percentage of isolates resistant to more than one antimicrobial (4.7%), followed by *Escherichia coli* isolates from birds (8.8%), *Enterococcus* isolates from bird feces (10.8%) and finally *Enterococcus* isolates from environmental samples (13.9%) with the highest level. Both genera were most commonly resistant to tetracycline combined with one or more of the other drugs tested (Tables 4 and 5).

**Table 4:** *Escherichia coli* isolate antimicrobial resistance patterns by site.

*N*	Control		Dairy		Beef A		Beef B		Antibiotics								
	Bird*	Environment	Bird	Environment	Bird	Environment	Bird	Environment	TE**	SXT	KF	EFT	AM	RD	AZM	AMC	C
1				1					R	R	R	R	R	S	S	R	R
1				1					R	R	I	S	R	R	S	S	R
1				1					R	S	R	S	R	S	S	S	R
1			1 (H)						R	S	S	S	R	S	S	S	R
1								1	R	R	S	S	S	S	S	S	S
2				1				1	R	S	S/I	S	S	R	S	S	S
1			1 (H)						R	S	S	S	R	S	S	S	S
7						3		4	R	S	S/I	S	S	S/I	S	S	S
28	1 (M)	3	3 (H)	5		6	1 (G)	9	S	S	S/I	S	S	R	S	S	S
1			1 (H)						S	S	R	S	S	S	S	S	S
1	1 (G)								S	R	S	S	S	S	S	S	S
66	9 (C, G, H-2,M-5)		10 (H-9,B)	7	11 (R-2, E-6, A-2, P)	18	4 (G, S, H, B)	7	S/I	S	S/I	S	S/I	S/I	S	S	S
111	11	3	16	16	11	27	5	22									

**Notes:**

** Table 1 shows antibiotic resistance combinations observed within individual isolates for all antibiotics tested. An “R” in the antibiotics column indicates the isolates listed by count (with bird species-number indicated in parentheses) was resistant to the antibiotics. An “S” indicates the all isolates in the row were susceptible to the antibiotic. An “I” indicates that all isolates in the row were of intermediate status. An “S/I” indicates that some of the isolates in that row were susceptible and at least one isolate had an intermediate phenotype. Antibiotic abbreviations: TE, tetracycline; SXT, trimethoprim-sulfamethoxazole; KF, cephalothin; EFT, ceftiofur; AM, ampicillin; RD, rifampin; AZM, azythromycin; AMC, ampicillin with clavulinate; C, chloramphenicol.

* Bird identity abbreviations: A, gray catbird; B, red-winged blackbird; C, brown-headed cowbird; G, common grackle; H, house sparrow; M, mourning dove; P, grasshopper sparrow; S, European starling.

**Table 5:** *Enterococcus* isolate antimicrobial resistance patterns by site and bacterial species.

		Control		Dairy		Beef A		Beef B		Antibiotics					
Species*	*N*	Bird**	Environment	Bird	Environment	Bird	Environment	Bird	Environment	TE***	SXT	CN	E	P	VA
C	1						1			R	S	S	R	S	S
C	2			1 (H)			1			R	S	S	I	S	S
C	1			1 (H)						S	S	R	I	S	S
C	1							1 (B)		S	S	I	I	S	R
C	18	4 (C-2,H,M)	1	1 (B)			6	1 (B)	5	S	S	S	S/I	S	S
D	6			1 (H)	1			1 (H)	3	R	S	S	S/I	S	S
D	1				1					S	S	I	R	S	S
D	7	1 (G)	1	1 (H)	4			1 (B, H)		S/I	S	S/I	S/I	S	S/I
Fs	1				1					R	R	R	I	S	R
Fs	1							1 (B)		R	R	R	R	S	I
Fs	3					2 (A, S)	1			R	S	R	R	S	S/I
Fs	1							1 (B)		S	S	R	R	S	R
Fs	2				1	1 (O)				R	S	S	R	S	S
Fs	1				1					S	S	S	R	S	R
Fs	1							1 (H)		S	S	R	I	S	R
Fs	4	1 (M)			1			1 (B)	1	R	S	S	S/I	S	S
Fs	2		1		1					S	S	I	I	S	R
Fs	7	1 (B)^&^	1	1 (H)				4 (B-3, S)		S	S	R	I	S	I
Fs	3				1			2 (B, H)		S	S	S/I	R	S	S
Fs	24		3	6 (H-5, B)	1	5 (R, S-4)	1	8 (R, C-2, G-2,H, B-2)		S	S	S/I	S/I	S	S/I
Fm	1				1					R	I	R	R	S	I
Fm	1			1 (H)						R	R	S	R	S	S
Fm	3			2 (H)	1					R	S	S	I	R	S
Fm	1								1	R	S	S	R	S	S
Fm	5			1 (B)^$^	4^$^					R	S	S	S/I	S	S
Fm	1			1 (H)						S	S	R	I	S	I
Fm	3							1 (H)	2	S	S	S	R	S	S
Fm	6			2 (H)	4					S	S	S	S/I	S	S
G	1							1 (B)		R	S	S	I	S	I
G	3			1 (H)	1				1	S	S	S	S/I	S	S/I
H	3						1		2	R	S	S	R	S	S
H	4	2 (C, M)		2 (H)						R	S	S	S/I	S	S
H	9			1 (H)	1		3		4	R	S	S/I	S/I	S	S
H	2						1		1^%^	S	S	S	R	S	S
H	32	12 (C-2, G, H-6, M-3)	5		2	2 (S)	2	5 (C-2, B-3)	4	S	S	S	S/I	S	S
All	162	21	12	23	26	10	17	29	24						

**Notes:**

* *Enterococcus* species abbreviations: c, *Enterococcus casseliflavus*; d, *Enterococcus durans*; fs, *Enterococcus faecalis*; fm, *Enterococcus faecium*; g, *Enterococcus gallinarum*; h, *Enterococcus hirae*.

** Bird identity abbreviations: A, gray catbird; B, red-winged blackbird; C, brown-headed cowbird; G, common grackle; H, house sparrow; M, mourning dove; O, song sparrow; P, grasshopper sparrow; R, American Robin; S, European starling.

*** Table 2 shows antibiotic resistance combinations observed within individual isolates for all antibiotics tested. An “R” in the antibiotics column indicates the isolates listed by count (with bird species-number indicated in parentheses) was resistant to the antibiotics. An “S” indicates the all isolates in the row were susceptible to the antibiotic. An “I” indicates that all isolates in the row were of intermediate status. An “S/I” indicates that some of the isolates in that row were susceptible and at least one isolate had an intermediate phenotype. Antibiotic abbreviations: TE, tetracycline; SXT, trimethoprim-sulfamethoxazole; CN, gentamicin; E, erythromycin; P, penicillin G; VA, vancomycin.

& SXT not tested for one isolate assigned to this profile due to laboratory error (one control house sparrow).

$ E not tested for two isolates assigned to this profile due to laboratory error (one each of a red-winged blackbird and environmental sample at the dairy site).

% TE and SXT not tested for one isolate assigned to this profile due to laboratory error (one environmental sample from beef R).

**Figure 2:**
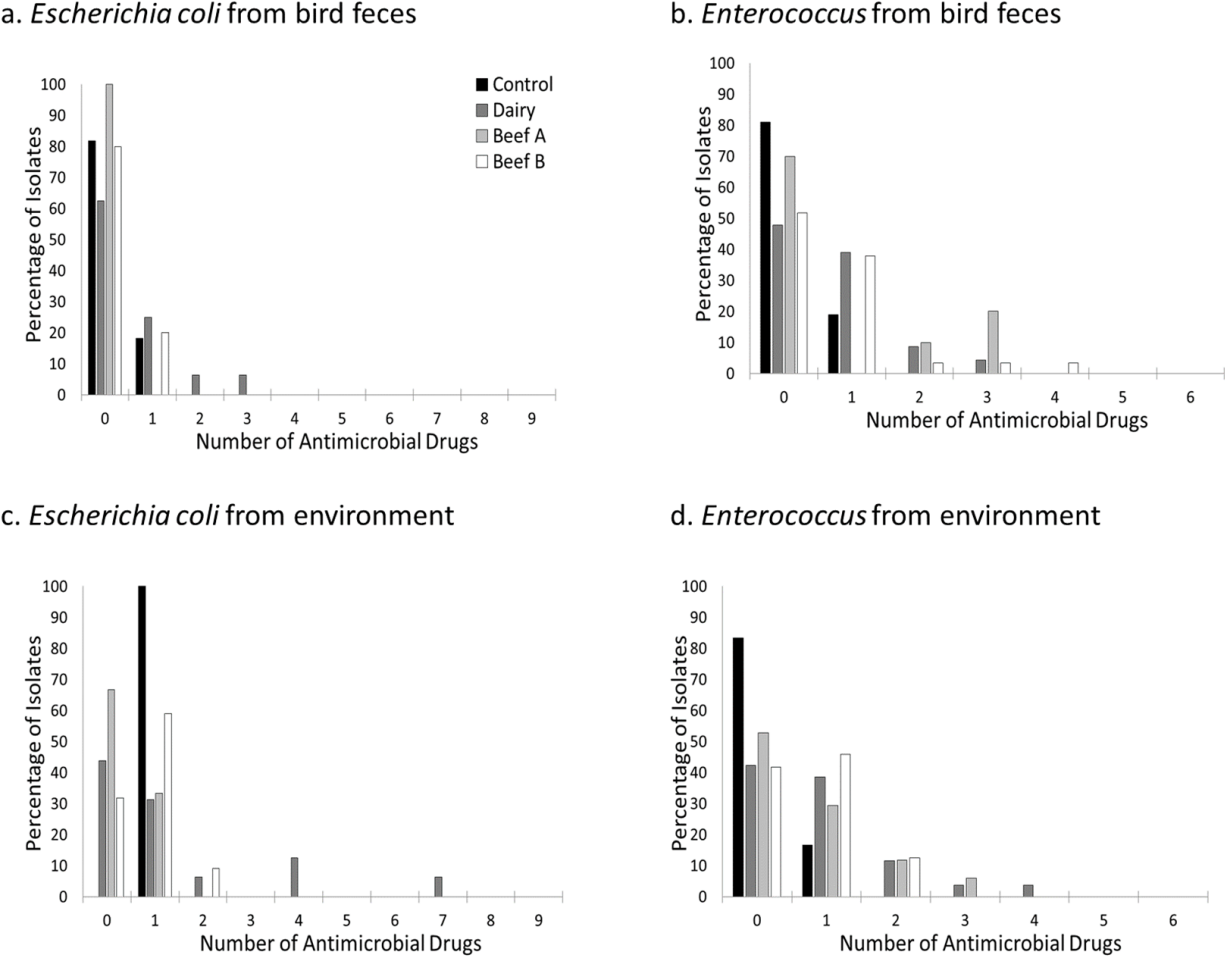
Prevalence of resistance to one or more antimicrobial drugs by site, bacterial genus, and isolate source. Percentage of *Escherichia coli* (A and C) and *Enterococcus* (B and D) isolates resistant to none, one, or more antimicrobial drugs at a residential control and three cattle farm sites in central Illinois by sample source. *Escherichia coli* resistant to more than one antimicrobial drug were primarily found in bird and environmental samples from the Dairy site. In contrast, *Enterococcus* isolates were seen in both sample types at all three farm facilities but less commonly at the Control.

**Figure 3:**
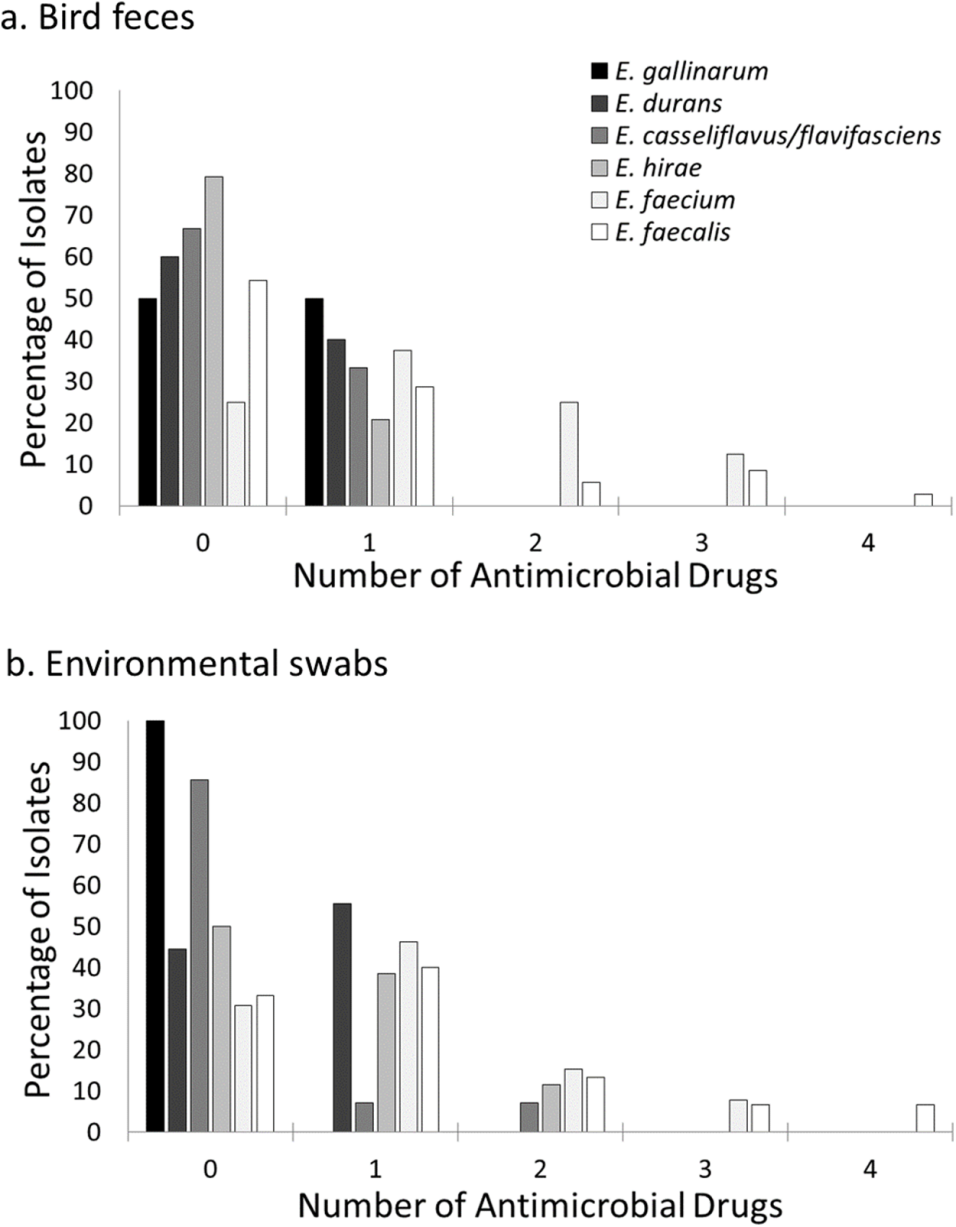
Prevalence of resistance to multiple antimicrobial drugs in *Enterococcus* species by site and isolate source. Percentage of Enterococcus isolates from bird feces (A) and environmental swabs (B) resistant to none, one or more antimicrobial drugs at a residential Control and three cattle farm sites in central Illinois. *Enterococcus faecalis* and *Enterococcus faecium* isolates were the majority of isolates with resistance to more than one antimicrobial drug for both sample types. A small number of *Enterococcus hirae* and *Enterococcus casseliflavus* isolates from environmental samples were resistant to two antimicrobial drugs.

The percentage of isolates resistant to one or more antimicrobial drugs was higher in environmental isolates compared to those derived from birds (51.0% vs 33.3% for all isolates), but this difference was more pronounced for *Enterococcus* isolates (59.4% vs 39.8%) compared to *Escherichia coli* isolates (52.9% vs 20.9%). Resistance to more than two antimicrobial drugs was relatively rare and primarily observed in *Escherichia coli* and *Enterococcus* isolates from the farm facilities (Fig. 2). For *Escherichia coli,* resistance to more than one antimicrobial drug was primarily detected in environmental isolates (majority at the Dairy and two isolates from Beef B), and just two bird isolates (both from house sparrows at the Dairy, Table 4). *Enterococcus faecium* and *Enterococcus faecalis* isolates demonstrating resistance to two or more antimicrobial drugs were cultivated from both bird feces and environmental swabs across all three farm sites but not from the control site (Fig. 3). *Enterococcus faecalis* resistant to more than one antimicrobial drug were isolated from birds on the Beef A and Beef B sites and *Enterococcus faecium* resistant to more than one antimicrobial drug were isolated from birds on the Dairy site. (Table 5).

### Multivariate modeling

Two multivariate models were developed to explore associations of sampling site, source, and bacterial genera to the outcome of antimicrobial drug resistance presence (resistance to one or more of the tested medication as a binomial outcome in a logistic model) and severity of resistance (the count of the number of antimicrobial drugs each isolate was resistant to as the outcome in a negative binomial model). The model including all isolates demonstrated significant differences by site and sample source for both presence of resistance and severity of multiple resistance (Table 6A). However, significant interaction terms with bacterial genus for both of these variables suggests that these differences vary in strength or direction between the two bacterial groups (Table 6A; Fig. 1).

**Table 6:** Multivariate models for antimicrobial drug resistance presence and severity of multiple resistance.

Model/effect	Psresence model d.f.	Presence model LR Chi-square	Presence model^&^ All isolates	Severity model d.f.	Severity model LR Chi-square	Severity model^&^ All isolates
A. All isolates						
Site	3	17.531	<0.001*	3	22.094	<0.001*
Source	1	8.606	0.003*	1	5.654	0.017*
Bacterial genus	1	0.539	0.463	1	0.375	0.540
Site:Source	3	2.090	0.554	3	0.038	0.998
Site:Bacterial genus	3	8.178	0.042*	3	13.314	0.004*
Source:Bacterial genus	1	6.479	0.011*	1	7.748	0.005*
Site:Source:Bacterial genus	3	4.861	0.182	3	3.138	0.371
B. *Escherichia coli*						
Site	3	11.329	0.010*	3	20.076	0.002*
Source	1	13.764	0.002*	1	13.536	0.002*
Site:Source	3	6.395	0.094**	3	2.677	0.444
C. *Enterococcus* genus						
Site	3	13.478	0.004*	3	15.854	0.001*
Source	1	0.931	0.335	1	0.111	0.740
Site:Source	3	0.556	0.906	3	0.483	0.923

**Notes:**

& Presence model was a logistic (binomial) model performed on a variable derived from observation of presence or absence of resistance to one or more antimicrobial drugs tested. Multiple resistance “severity” model was a negative binomial (count) model performed on a variables derived from observation of the number of antimicrobial drugs to which each isolate demonstrated resistance.

* Significant at α = 0.05.

** Marginally significant at α = 0.10.

To explore the significant interaction terms in the full model, individual models were run for each bacterial genus. For *Escherichia coli* isolates, both site and source were significant factors associated with presence of resistance and severity of resistance (Table 6B; Fig. 2). In contrast, *Enterococcus* isolates varied significantly only by sampling site for both presence of resistance and severity of resistance when examined at the genus level (Table 6C).

### *Escherichia coli* genetics

**Figure 4:**
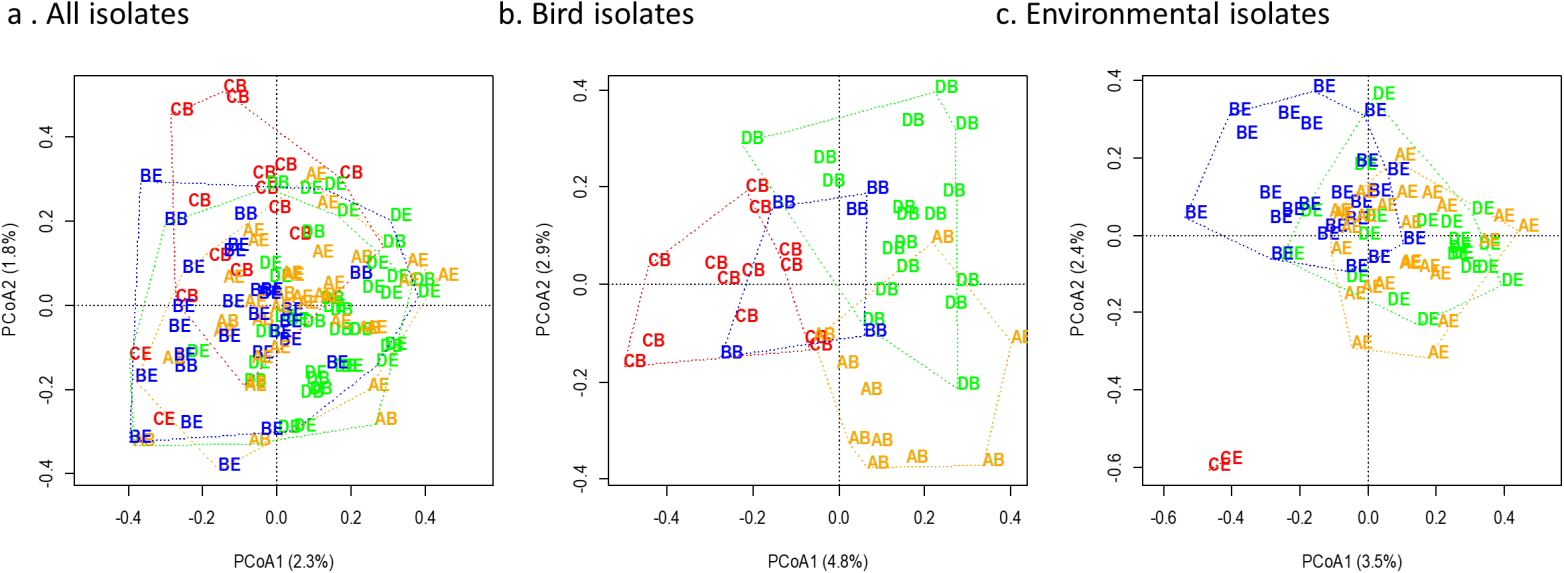
Genomic diversity of *Escherichia coli* by site and isolate source. Genomic diversity of *Escherichia coli* at a residential control and three cattle farm sites in central Illinois by site for both sources (A), from just bird feces (B), and from just environmental swabs (C). Sample points are labelled by site and source and colored by site. The first letter indicates source: D, Dairy (green); A, Beef A (orange); B, Beef B (blue), and C, Control (red). The second letter indicates sample source: B, bird feces or swab; E, environmental swab.

Overall, both sampling site and sample source were significant factors impacting similarity of *Escherichia coli* genomic fingerprint patterns, with a marginal interaction term (Permutation MANOVA: Source *p* = 0.021, Site *p* < 0.001, Source*Site *p* = 0.073). When isolates were evaluated by sample sources (bird feces or environmental swabs) independently, sampling site was significantly associated with genomic similarity for both the environmental and bird-derived isolates (Permutation MANOVA: bird isolates *p* = 0.013, environmental *p* < 0.001 with all sites, *p* = 0.010 when Control is omitted due to small sample size (*n* = 2)). The degree of separation among sites was more notable for bird-derived isolates compared to those from environmental sources when visualized by constrained principal coordinates analysis (Fig. 4). Notably, while the two *Escherichia coli* isolates sourced from the Control environment were highly dissimilar to those from the farm environments, the farm-derived isolates showed more overlap in site-based clusters compared to isolates obtained from bird feces.

## Discussion

The presence of similarities in the antimicrobial resistance profiles of *Escherichia coli* and *Enterococcus* species in isolates from birds and farm environments could suggest that bacterial populations carried by birds are linked to environmental exposures on a relatively local level. However, the strength of the observed linkages differed by bacterial group, a finding which could affect capacity to reliably use different target organisms to detect subtle associations of microbial exchange with factors such as geography, landscape variables, host species identity, or farm management approaches.

*Escherichia coli* was isolated at moderate prevalence in wild bird feces, even when there was ample opportunity for exposure in the farm environment. Further, antimicrobial resistance was not as commonly detected in isolates from birds at the control and beef facilities compared to those captured at the intensive dairy facility. However, genomic fingerprinting analysis of our *Escherichia coli* isolates suggests that while bird populations do share a local bacterial pool with the farm environment in which they were captured, they also may carry isolates circulating more regionally that may reflect time spent in other non-farm habitats.

We also considered if one or more *Enterococcus* species might be an alternative model organism for considering microbial transmission dynamics between species in farm environments. Most birds carried at least one species of *Enterococcus*, with a subset of species being particularly common (notably *Enterococcus faecalis, Enterococcus faecium* and *Enterococcus hirae*). While not directly examined in this study, it is likely that many individuals carry more than one *Enterococcus* species such that targeted isolation would further increase detected prevalence of these species. Plates examined for growth of presumptive *Enterococcus* isolates frequently demonstrated more than one colony phenotype, although only one colony of the dominant phenotype was arbitrarily selected for identification and analysis in this study (E.W. Lankau, 2009, personal observation). The overall pattern of *Enterococcus* antimicrobial resistance in bird-derived isolates more strongly reflected the species and bacterial pools of the sites where they were captured. These results suggest that study of the *Enterococcus* community or targeted cultivation of a single *Enterococcus* species as a model organism could provide sufficient numbers of isolates from a relatively small sample of birds from multiple sites to support development of causal models of microbial exchange between birds and livestock in farm environments.

*Enterococcus* species and antimicrobial resistance traits of isolates carried by wild passerine birds in agricultural landscapes have not been extensively studied, but our results are compatible with similar studies in wild passerines (Radimersky et al., 2010; Santos et al., 2013) and parallel findings in wild and captive raptors from the same geographic region and time period as our study (Marrow et al., 2009). Wild raptors from central Illinois, which might regularly consume passerine species that frequent farms, commonly carried *Enterococcus* species, particularly *Enterococcus faecalis,* and these isolates demonstrated resistance to a number of antimicrobial drugs classes tested in common with the present study (Marrow et al., 2009). A study that documented antimicrobial resistance across a number of bacterial taxa isolated from wild birds in Michigan showed a similarly higher prevalence of resistance in *Enterococcus* isolates relative to a number of other taxa but did not detect differences in the overall prevalence of resistance among landscape types (Carter et al., 2018).

Genomic fingerprinting was not performed on *Enterococcus* isolates in this study due to resource constraints. However, work in the source-tracking field suggests that enterococci may be a more powerful model than *Escherichia coli* for more finely discerning origins by comparison of molecular fingerprint to established libraries because environmental isolates of *Enterococcus* species are not limited to animal source (Hassan, Ellender & Wang, 2007). Although molecular fingerprinting was not performed on the *Enterococcus* isolates in the present study, *Enterococcus* species were more frequently isolated from the residential control environment, providing a more statistically robust sample for comparing bird and environmental isolates by phenotypic or genetic means. Only three isolates of *Escherichia coli* were obtained from the residential control environment for comparison to bird-origin isolates.

## Limitations

This study was not designed to elucidate causes or mechanisms of antimicrobial resistance transmission to or by wild birds. Samples were collected across two seasons (fall and early summer) that may have distinct ecological effects on bird behavior and microbial exposures in the farm setting. Neither sample size, nor diversity of farms studied is sufficient for this purpose and the spatial proximity and common management of the university-owned farms presents an additional confounding factor. Further, different bird species may interface with the same habitat differently due to feeding, nesting, or roosting behaviors that vary their actual microbial exposures (Carter et al., 2018) and bird diversity was not controlled for in this study. Rather, the goal was to explore how choice of model bacterium might impact strength of findings and resulting management recommendations. In addition, this study only considered two bacterial genera selected due to clinical interest and ease of cultivation, but other bacterial groups may more accurately reflect microbial exchange dynamics or capture different nuances of these exchanges.

Bird species diversity (both species observed by the researchers and those captured during sampling) differed among sites, which may also introduce a potential source of variability that prohibits interpreting specific differences between the three livestock facilities. Bird species diversity is a variable that would be best controlled for in future studies, although this may be challenging as birds captured at different locations may reflect real bird community differences or differences in habitat use or behavior among species that may also be an important contributing factor for determining bacterial transmission patterns within and among facilities.

Finally, antimicrobial resistance was used as a phenotypic measure of association between bird and environmental bacterial isolates that may reflect both direct lineage and horizontal transfer of resistance traits. Community level cultivation or next-generation sequencing methods for documenting total microbial community differences and antibiotic resistance genetics (i.e., the “resistome”) in environmental or fecal samples will be important for applying causal reasoning to inferring the direction and rate of bacterial exchange in farm environments.

## Conclusions

This study supports the generally accepted idea that wild bird populations residing on livestock facilities may acquire bacteria from farm exposures and could contribute to short-distance spread of pathogens and antimicrobial resistant bacteria (Bonnedahl & Järhult, 2014), but also considers how to select a model for exploring the dynamics and directionality of microbial exchange more generally. Selection of a bacterial model that is of relatively high prevalence and that strongly reflects environmental influences on community membership might facilitate application of landscape ecology approaches to studying the role of wild birds in antimicrobial resistance spread. Such landscape level studies have great potential for teasing apart the role of selective pressures within the intestinal tract of host species for modifying the ecology of antimicrobial resistant strains (Singer, Ward & Maldonado, 2006). Similarly, prospective studies with increased farm, bird species, and geographic diversity are needed to understand factors such as strain turnover rate and retention of environmentally-derived bacteria that might modulate the impacts of wild birds and other wildlife species pose on agricultural biosecurity and food safety (Greig et al., 2015). Improved understanding of farm management practices that reduce microbial exchange between wildlife and livestock could mitigate any potential contributions to disease transmission while also protecting the health and stability of wildlife populations.

Finally, pairing focal studies of antimicrobial resistance in specific model bacterial species with approaches to assessing microbial resistance at a community level (i.e., molecular or phenotypic assessment of the community “resistome”) could provide important context for understanding the ecology of antimicrobial resistance gene transmission within and among bacterial taxa that birds encounter across diverse landscapes (Carter et al., 2018). Such studies could aid in understanding microbial exchange on and among farm environments and support development of wildlife conservation-oriented management strategies on farms that protect livestock, wildlife, and surrounding communities.

## Supplemental Information

10.7717/peerj.6460/supp-1 Supplemental Information 1 Table S1. Concentrations and diffusion zone breakpoints for resistance to antimicrobial drugs tested in this study.

10.7717/peerj.6460/supp-2 Supplemental Information 2 Table S2. Presence of three bacterial genera in wild bird feces and cattle farm environments in central Illinois. Raw data.

10.7717/peerj.6460/supp-3 Supplemental Information 3 Table S3. Antimicrobial disc diffusion analysis of enteric bacteria isolated from wild bird feces and cattle farm environments in central Illinois. Raw data.

10.7717/peerj.6460/supp-4 Supplemental Information 4 Table S4. Molecular fingerprint bands for *Escherichia coli* isolated from wild bird feces and cattle farm environments in central Illinois. Raw data.

10.7717/peerj.6460/supp-5 Supplemental Information 5 Table S5. Metadata for three datasets describing bacterial prevalence, antimicrobial resistance, and genomic fingerprint relationships of enteric bacteria isolated from wild birds and cattle farm environments in central Illinois. Raw data.

10.7717/peerj.6460/supp-6 Supplemental Information 6 Table S6. Bird species captured by sampling site.

## Additional Information and Declarations

### Field Study Permissions

The following information was supplied relating to field study approvals (i.e., approving body and any reference numbers): All capture and handling was performed as a sub-permitee of the Illinois Natural History Survey’s master banding permit (#06507).

### Data Availability

The following information was supplied regarding data availability: Data and data dictionary are available as Supplemental Files (Tables S3–S6). These data include microbial prevalence, antimicrobial resistance phenotypes and *Escherichia coli* fingerprinting data along with metadata describing the included variables.

## References

[ref-1] Abulreesh H, Goulder R, Scott G (2007). Wild bird and human pathogens in the context of ringing and migration. Ringing and Migration.

[ref-2] Arnold KE, Williams NJ, Bennett M (2016). ‘Disperse abroad in the land’: the role of wildlife in the dissemination of antimicrobial resistance. Biology Letters.

[ref-3] Bonnedahl J, Järhult JD (2014). Antibiotic resistance in wild birds. Upsala Journal of Medical Science.

[ref-4] Brittingham MC, Temple SA, Duncan RM (1988). A survey of the prevalence of selected bacteria in wild birds. Journal of Wildlife Diseases.

[ref-5] Burton PJ, Thornsberry C, Yee YC, Watts JL, Yancey RJ (1996). Interpretive criteria for antimicrobial susceptibility testing of ceftiofur against bacteria associated with swine respiratory disease. Journal of Veterinary Diagnostic Investigation.

[ref-6] Carter DL, Docherty KM, Gill SA, Baker K, Teachout J, Vonhof MJ (2018). Antibiotic resistant bacteria are widespread in songbirds across rural and urban environments. Science of the Total Environment.

[ref-7] Colston TJ, Jackson CR (2016). Microbiome evolution along divergent branches of the vertebrate tree of life: what is known and what is unknown. Molecular Ecology.

[ref-8] Galland J, Hyatt D, Crupper S, Acheson D (2001). Prevalence, antibiotic susceptibility, and diversity of *Echerichia coli* O157:H7 isolates from a longitudinal study of beef cattle feedlots. Applied and Environmental Microbiology.

[ref-9] Goldberg TL, Gillespie TR, Rwego IB, Wheeler E, Estoff EL, Chapman CA (2007). Patterns of gastrointestinal bacterial exchange between chimpanzees and humans involved in research and tourism in western Uganda. Biological Conservation.

[ref-10] Greig J, Raji A, Young I, Mascarenhas M, Waddell L, LeJeune J (2015). A scoping review of the role of wildlife in the transmission of bacterial pathogens and antimicrobial resistance to the food chain. Zoonoses and Public Health.

[ref-11] Hassan WM, Ellender RD, Wang SY (2007). Fidelity of bacterial source tracking: *Escherichia coli* vs. *Enterococcus* spp and minimizing assignment of isolates from nonlibrary sources. Journal of Applied Microbiology.

[ref-12] Hong PY, Wheeler E, Cann IKO, Mackie RI (2011). Phylogenetic analysis of the intestinal microbial community in herbivorous land and marine iguanas of the Galápagos Islands using 16S rRNA-based pyrosequencing. ISME Journal.

[ref-13] Humphry RW, Cameron A, Gunn GJ (2004). A practical approach to calculate sample size for herd prevalence surveys. Preventive Veterinary Medicine.

[ref-14] Jackson CR, Fedorka-Cray PJ, Barrett JB (2004). Use of a genus- and species-specific multiplex PCR for identification of *Enterococci*. Journal of Clinical Microbiology.

[ref-15] Marrow J, Whittington JK, Mitchell M, Hoyer LL, Maddox C (2009). Prevalence and antibiotic-resistance characteristics of *Enterococcus* spp. isolated from free-living and captive raptors in Central Illinois. Journal of Wildlife Diseases.

[ref-16] McCord AI, Chapman CA, Weny G, Tumukunde A, Hyeroba D, Klotz K, Koblings AS, Mbora DNM, Cregger M, White BA, Leigh SR, Goldberg TL (2013). Fecal microbiomes of non-human primates in Western Uganda reveal species-specific communities largely resistant to habitat perturbation. American Journal of Primatology.

[ref-17] Murray PR, Baron EJ, Jorgensen JH, Landry ML, Pfaller MA (2007). Manual of clinical microbiology.

[ref-18] National Committee for Clinical Laboratory Standards (NCCLS) (2003). M2-A8 Performance standards for antimicrobial disc susceptibility tests (M2-A8) and Disc Diffusion Supplemental Tables (M100-S13[M2]).

[ref-19] Oksanen J, Blanchet FG, Friendly M, Kindt R, Legendre P, McGlinn D, Minchin PR, O’Hara PB, Simpson GL, Solymos P, Stevens MHH, Szoecs E, Wagner H (2018). http://CRAN.R-project.org/package=vegan.

[ref-20] Pesapane R, Ponder M, Alexander KA (2013). Tracking pathogen transmission at the human-wildlife interface: banded mongoose and *Escherichia coli*. Ecohealth.

[ref-21] Poyart C, Quesnes G, Trieu-Cout P (2000). Sequencing the gene encoding manganese-dependent superoxide dismutase for rapid species identification of *Enterococci*. Journal of Clinical Microbiology.

[ref-22] Rademaker JL, Louws W, Louws FJ, De Bruijn FJ (1998). Characterization of the diversity of ecologically important microbes by rep-PCR genomic fingerprinting. Molecular Microbial Ecology Manual.

[ref-23] Radimersky T, Frolkova P, Janoszowska D, Dolejska M, Svec P, Roubalova E, Cikova P, Cizek A, Literak I (2010). Antibiotic resistance in faecal bacteria (*Escherichia coli*, *Enterococcus* spp.) in feral pigeons. Journal of Applied Microbiology.

[ref-24] R Core Team (2017). R: a language and environment for statistical computing.

[ref-25] Risely A, Waite DW, Ujvari B, Hoye BJ, Klaassen M (2017a). Active migration is associated with specific and consistent changes to gut microbiota in *Calidris* shorebirds. Journal of Animal Ecology.

[ref-26] Risely A, Waite DW, Ujvari B, Klaassen M, Hoye B (2017b). Gut microbiota of a long-distance migrant demonstrates resistance against environmental microbe incursions. Molecular Ecology.

[ref-27] Rwego IB, Isabirye-Basuta G, Gillespie TR, Goldberg TL (2008). Gastrointestinal bacterial transmission among humans, mountain gorillas, and livestock in Bwindi Impenetrable National Park, Uganda. Conservation Biology.

[ref-28] Santos T, Silva N, Igrejas G, Rodrigues P, Micael J, Rodrigues T, Resendes R, Gonçcalves A, Marinho C, Gonçcalves D, Cunha R, Poeta P (2013). Dissemination of antibiotic resistant *Enterococcus* spp. and *Escherichia coli* from wild birds of Azores Archipelago. Anaerobe.

[ref-29] Singer R, Ward M, Maldonado G (2006). Can landscape ecology untangle the complexity of antibiotic resistance?. Nature Reviews Microbiology.

[ref-30] US Department of Agriculture (2015). Environmental assessment: bird damage management in the state of Illinois. https://www.aphis.usda.gov/wildlife_damage/downloads/nepa/2015%20EA%20Bird%20Damage%20Management%20in%20Illinois.pdf.

[ref-31] VanderWaal KL, Atwill ER, Isbell LA, McCowan B (2013). Quantifying microbe transmission networks for wild and domestic ungulates in Kenya. Biological Conservation.

[ref-32] Venables WN, Ripley BD (2002). Modern applied statistics with S.

[ref-33] Versalovic J, Koeuth T, Lupski JR (1991). Distribution of repetitive DNA sequences in eubacteria and application to fingerprinting of bacterial genomes. Nucleic Acids Research.

[ref-34] Vittecoq M, Gadreuil S, Prugnolle F, Durand P, Brazier L, Renaud N, Arnal A, Aberkane S, Jean-Pierre H, Gauthier-Clerc M, Thomas F, Renaud F (2016). Antimicrobial resistance in wildlife. Journal of Applied Ecology.

[ref-35] Waite DW, Taylor MW (2014). Characterizing the avian gut microbiota: membership, driving influences, and potential function. Frontiers in Microbiology.

[ref-36] Wheeler E, Hong PY, Bedon LC, Mackie RI (2012). Carriage of antibiotic-resistant enteric bacteria varies among sites in Galápagos reptiles. Journal of Wildlife Diseases.

