# Supplementary material for: Considerations for studying transmission of antimicrobial resistant enteric bacteria between wild birds and the environment on intensive dairy and beef cattle operations
Source: PeerJ. 2019 Feb 27;7:e6460. doi: 10.7717/peerj.6460 (PMC6397636; doi:10.7717/peerj.6460)
Supplement: Supplemental Information 1 [file peerj-07-6460-s001.docx]

Table S1. Concentrations and diffusion zone breakpoints for resistance to antimicrobial drugs tested in this study

| **Drug class** | **Antimicrobial drug*** | **Drug quantity/disc** | **Inhibition zone breakpoints for analysis R, I,S (mm)** | **Bacteria tested** |
| --- | --- | --- | --- | --- |
| Aminoglycoside | Gentamicin | 10 μg | ≤14,-, ≥15 | *Enterococcus* |
| Ansamycin | Rifampin | 5 μg | ≤8,9, ≥10** | *E. coli* |
| Cephalosporin | Cephalothin | 30 μg | ≤14,15-17, ≥18 | *E. coli* |
| Cephalosporin (veterinary) | Ceftiofur | 30 μg | ≤17,18-20, ≥21 | *E. coli* |
| Folate pathway inhibitor | Sulfamethoxazole-trimethoprim 19:1 | 23.75/1.25 μg | ≤10,11-15,>16 | Both |
| Glycopeptide | Vancomycin | 30 μg | ≤14,15-16, ≥17 | *Enterococcus* |
| Macrolide | Azithromycin | 15 μg | ≤18,-, ≥18 | *E. coli* |
| Macrolide | Erythromycin | 15 μg | ≤13,14-22, ≥23 | *Enterococcus* |
| Penicillin | Ampicillin | 10 μg | ≤13,14-16, ≥17 | *E. coli* |
| Penicillin | Penicillin G | 10 units | ≤14,15-18, ≥19 | *Enterococcus* |
| β-lactam- β-lactamase inhibitor | Amoxicillin-clavulanic acid | 20/10 μg | ≤14,15-16, ≥17 | *E. coli* |
| Phenicol | Chloramphenicol | 30 μg | ≤12,13-17, ≥18 | *E. coli* |
| Tetracycline | Tetracycline | 30 μg | ≤14,15-18,>19 | Both |

*Antimicrobial disc supplier: BD BBL™ Sensi-Disc™ Susceptibility Test Discs, Becton, Dickinson, and Company, Franklin Lakes, NJ

** Clinical resistance thresholds are not defined for rifampin in members of Enterobacteriaceae. The normal diameter range for quality control using *E. coli* ATCC 25922 (8-10 mm) was used to indicate isolates with near absolute resistance to rifampicin versus organisms with larger zones of inhibition, however, these arbitrary thresholds do not have clinical relevance for treatment as rifampin is generally not recommended for *E. coli* infections. This was done purely as a phenotypic categorization used for purposes of data analysis with no regard to clinical implications. Breakpoints for *Staphylococcus* and *Enterococcus* species are ≤16mm,17-19 mm, and ≥20 mm for resistant, intermediate and susceptible, respectively, whereas all *E. coli* isolates examined in this study had zones of inhibition less than 17 mm (range: growth adjacent to the disc at ~7mm-17mm).
