# Supplementary material for: Considerations for studying transmission of antimicrobial resistant enteric bacteria between wild birds and the environment on intensive dairy and beef cattle operations
Source: PeerJ. 2019 Feb 27;7:e6460. doi: 10.7717/peerj.6460 (PMC6397636; doi:10.7717/peerj.6460)
Supplement: Supplemental Information 6 [file peerj-07-6460-s006.docx]

Table S2. Bird species captured by sampling site

| **Bird species** | **Control**  **n (col%)** | **Dairy**  **n (col%)** | **Beef A**  **n (col%)** | **Beef B**  **n (col%)** | **Total**  **n (col%)** | ***E. coli* prevalence** | ***Enterococcus* prevalence** | ***S. enterica***  **prevalence** |
| --- | --- | --- | --- | --- | --- | --- | --- | --- |
| House sparrow | 12 (40.0%) | 30 (78.9%) | - | 6 (20.0%) | 48 (37.5%) | 41.7% | 72.9% | 2.1% |
| Red-winged black bird | - | 5 (13.2%) | 1 (3.3%) | 15 (50.0%) | 21 (16.4%) | 9.5% | 85.7% | 4.8% |
| European starling | - | 1 (2.6%) | 17 (56.7%) | 1 (3.3%) | 19 (14.8%) | 36.8% | 94.7% | 0% |
| Brown-headed cowbird | 6 (20.0%) | 2 (5.3%) | - | 5 (16.7%) | 13 (10.2%) | 7.7% | 69.2% | 0% |
| Mourning dove | 10 (33.3%) | - | - | - | 10 (7.8%) | 80% | 60% | 0% |
| American robin | - | - | 3 (10%) | 1 (3.3%) | 4 (3.1%) | 50% | 100% | 0% |
| Common grackle | 2 (6.7%) | - | - | 2 (6.7%) | 4 (3.1%) | 100% | 100% | 0% |
| Gray catbird | - | - | 2 (6.7%) | - | 2 (1.6%) | 100% | 100% | 0% |
| Grasshopper sparrow | - | - | 2 (6.7%) | - | 2 (1.6%) | 50% | 100% | 0% |
| Song sparrow | - | - | 2 (6.7%) | - | 2 (1.6%) | 0% | 50% | 0% |
| American goldfinch | - | - | 1 (3.3%) | - | 1 (0.8%) | 0% | 0% | 0% |
| Barn swallow | - | - | 1 (3.3%) | - | 1 (0.8%) | 0% | 100% | 0% |
| Eastern meadowlark | - | - | 1 (3.3%) | - | 1 (0.8%) | 0% | 100% | 0% |
| **Total** | **30** | **38** | **30** | **30** | **128** | **36.7%** | **81.5%** | **1.6%** |
